# Diagnostic value of heparin-binding protein in the cerebrospinal fluid for purulent meningitis in children

**DOI:** 10.1590/1414-431X2021e11295

**Published:** 2021-09-03

**Authors:** Dan Ren, Di Wu, Fu Liu, Shuli Jiao, Yi Wu

**Affiliations:** 1 Department of Pediatrics, Mianyang Central Hospital, Mianyang, Sichuan, China; 2 Department of Gynecology and Pediatrics, Hospital of PLA Unit 63820, Mianyang, Sichuan, China

**Keywords:** Pyogenic meningitis, HBP, CRP, PCT, TNF-α

## Abstract

This study aimed to investigate the diagnostic value of heparin-binding protein (HBP) in the cerebrospinal fluid of children with purulent meningitis (PM). This study included 118 children with PM diagnosed at our hospital from January 2018 to January 2020, 110 children with viral meningitis (VM) and 80 children with suspected meningitis who were ruled out by cerebrospinal fluid (CSF) analysis during the same period. HBP and white blood cell (WBC) count in the CSF, and inflammatory factors, including C-reactive protein (CRP), tumor necrosis factor (TNF)-α, and procalcitonin (PCT), were measured. Receiver-operator characteristic curves were used to analyze the predictive value of HBP, CRP, PCT, and TNF-α levels in the diagnosis of PM by CSF analysis. HBP levels in the CSF of children with PM were higher, while the CRP and serum PCT and TNF-α levels were elevated in all groups (P<0.05). In addition, HBP levels in the CSF were more accurate for the diagnosis of PM than traditional diagnostic indexes. HBP levels in the CSF can be used as an important reference for early diagnosis of PM.

## Introduction

Purulent meningitis (PM) is an infection of the meninges caused by a purulent bacterial infection and a common purulent infection of the central nervous system. It is more common in children, and some children can show a severe condition and die within 24 h if they are not treated in time. Frequently showing high fever, headache, vomiting, severe malaise, disturbance of consciousness, occasional convulsions, oliguria, or anuria, patients suffering from brain parenchymal damage quickly enter a coma, with frequent convulsions, hemiplegia, high blood pressure, dilated pupils on one side, lack of light reflex sign, and fixed eyeballs followed by respiratory failure and death. The incidence of PM has been increasing with the misuse of antibiotics (1), and the mortality rate has been reported to be >40% in children (2). Diagnosis of the disease is difficult to confirm due to the lack of apparent specific early clinical manifestations, which contributes to a higher rate of disability (3) and seriously affects the daily life of patients and their families. Currently, the gold standard for the diagnosis of PM is cerebrospinal fluid (CSF) analysis (4). However, the long testing time and low accuracy have limited its clinical application. Therefore, early diagnosis of PM is crucial. A study found that C-reactive protein (CRP), tumor necrosis factor (TNF)-α, and procalcitonin (PCT) levels can be potential markers. However, their application in clinical diagnosis has some limitations (5).

Heparin-binding protein (HBP), which has azurophilic granules and is released by secretory vesicles of neutrophils, is a serine protease (6). It has been shown that HBP is involved in the development of a variety of inflammatory conditions and infections (7). In the early stages of the inflammatory process, eosinophilic granules and secretory vesicles secrete HBP, which acts by activating various types of cells (8), including monocytes, and leads to vascular leakage and edema formation (9). It has been shown that HBP is also secreted after polymorphonuclear cell exudation and promotes inflammatory responses (10). In a study from 2011, Linder et al. (11) describes HBP as a superior marker of bacterial meningitis in adults. The association between HBP levels in the CSF and the diagnosis of PM in children has not been reported. This study assessed the diagnostic value of HBP in patients with PM, as well as non-PM, by comparing the expression levels of HBP with other biomarkers.

## Material and Methods

### Study subjects

A total of 308 children who visited our hospital between January 2018 and January 2020 were enrolled in this study [118 children with PM (PM group), 80 children initially suspected of having meningitis but whose diagnosis was ruled out (CON group), and 110 children with viral meningitis (VM group)]. All patients’ families were informed of the risks and signed informed consent forms. The ethics committee of Mianyang Central Hospital approved this study (ChiCTR1800015276). Children with PM and VM were selected strictly according to the inclusion and exclusion criteria. The inclusion criteria for PM were i) children with common symptoms of PM, including fever, irritability, vomiting, drowsiness, and impaired consciousness, and ii) positive bacterial cultures or smear test of the CSF. The inclusion criteria for VM were i) children with a final diagnosis of VM based on electroencephalogram, magnetic resonance imaging features, and serological tests, and ii) no detection of bacteria in the CSF culture or negative results in the smear test.

The exclusion criteria were i) children with congenital neurological malformations, ii) children with intracranial hemorrhage and septic infection, and iii) children with inherited metabolic disorders.

### Data collection

The enrolled patients underwent an initial clinical assessment, including demographics, vital signs (heart rate, respiratory rate, blood pressure, and arterial oxygen saturation), and history of antibiotic administration. Routine CSF tests (including HBP) and routine serum tests (including CRP, TNF-α, and PCT levels) were performed at hospital admission and at the start of effective treatment.

### Blood samples and laboratory analysis

After patients were admitted to the hospital, early morning fasting venous blood was collected, placed in anticoagulated tubes, and centrifuged at 99 *g* and 22°C for 10 min, and the supernatant was stored at -80°C. HBP levels in the CSF were determined using ELISA (Nanjing Xinfan Technology Co., Ltd., China). CRP was quantified by enzyme-linked fluorescence, PCT by latex immunoturbidimetric assay, and TNF-α levels by ELISA (Nanjing Xinfan Technology Co., Ltd.).

### Statistical analysis

Data were analyzed using SPSS software system version 23.0 (SPSS, USA) and GraphPad Prism V.8.0 (GraphPad Software, USA). One-factor ANOVA was used to compare the three groups. Correlation analysis was performed using a Spearman test. The diagnostic ability of HBP in the CSF was assessed using the area under the receiver operating characteristic curve (AUC). The significance level was set at α=0.05.

## Results

### Baseline data

The PM group included a total of 118 PM patients admitted to our hospital, the VM group included 110 VM patients and the CON group included 80 children with suspected meningitis, which was ruled out. Vital signs among all groups were not significantly different (Table 1).

**Table 1:** Baseline data.

	Control group	Viral meningitis group	Purulent meningitis group	P value
Case (n)	80	110	118	
Age (years)	3.2±0.6	3.7±0.5	3.6±0.4	0.667
Men/women (n)	41/39	63/47	60/58	0.784
Weight (kg)	18.9±2.1	20.3±2.1	20.1±2.2	0.731
Heart rate (per min)	89.7±1.6	89.2±1.7	88.4±1.6	0.548
Respiratory rate (per min)	20.2±1.1	19.8±1.1	21.0±1.3	0.872

Data are reported as means±SD or total number (ANOVA).

### Comparison of HBP levels in the CSF

**Figure 1:**
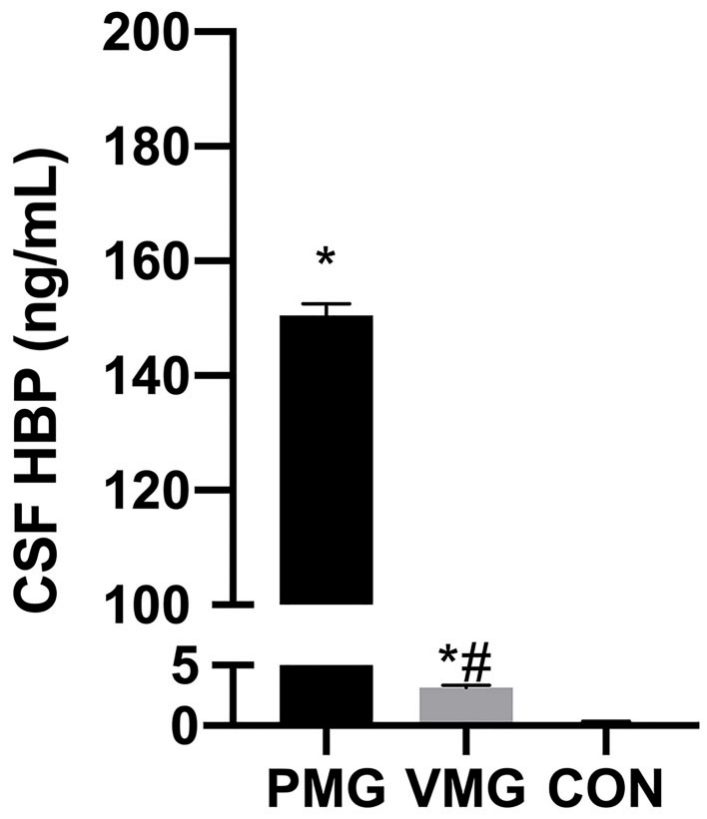
Comparison of heparin-binding protein (HBP) levels in the cerebrospinal fluid (CSF) of children. VMG: viral meningitis group; PMG: purulent meningitis group; CON: control group. Data are reported as means±SD. *P<0.05, *vs* CON; ^#^P<0.05, *vs* PMG (ANOVA).

HBP of the PM group was 150.52±1.87 ng/mL, of the VM group was 3.14±0.23 ng/mL, and of the CON group was 0.33±0.02 ng/mL. HBP levels were higher in the CSF of the PM group than in that of the CON and VM groups (P<0.05, Figure 1), indicating that HBP levels in the CSF could be used as a potential indicator for the diagnosis of PM.

**Figure 2:**
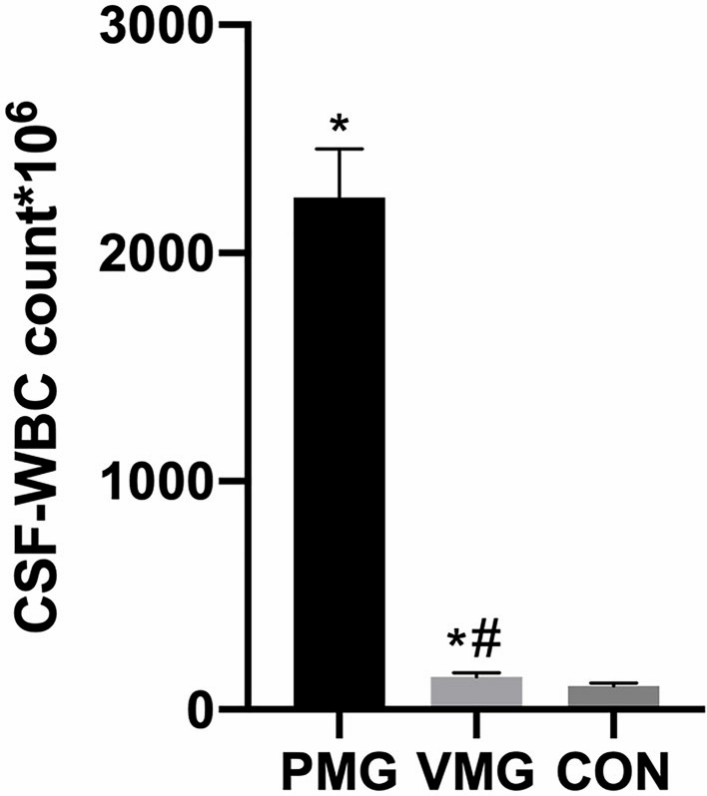
Comparison of cerebrospinal fluid white blood cell (CSF-WBC) count in the children. VMG: viral meningitis group; PMG: purulent meningitis group; CON: control group. Data are reported as means±SD. *P<0.05, *vs* CON; ^#^P<0.05, *vs* PMG (ANOVA).

White blood cell (WBC) count of the PM group was 2243±213 ng/mL, of the VM group was 143±18.43 ng/mL, and of the CON group was 104±1.22 ng/mL. CSF-WBC levels were higher in the PM group than in the CON and VM groups (P<0.05, Figure 2), indicating that CSF-WBC levels could be used as an indicator for the diagnosis of PM.

### Serum CRP, PCT, and TNF-α levels

**Figure 3:**
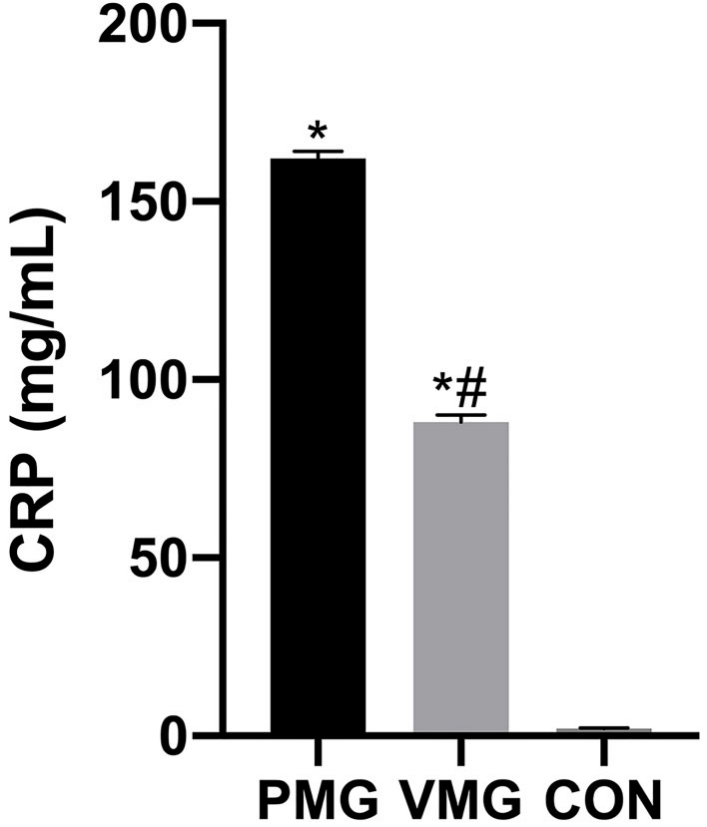
Comparison of serum C-reactive protein (CRP) levels of children. VMG: viral meningitis group; PMG: purulent meningitis group; CON: control group. Data are reported as means±SD. *P<0.05, *vs* CON; ^#^P<0.05, *vs* PMG (ANOVA).

**Figure 4:**
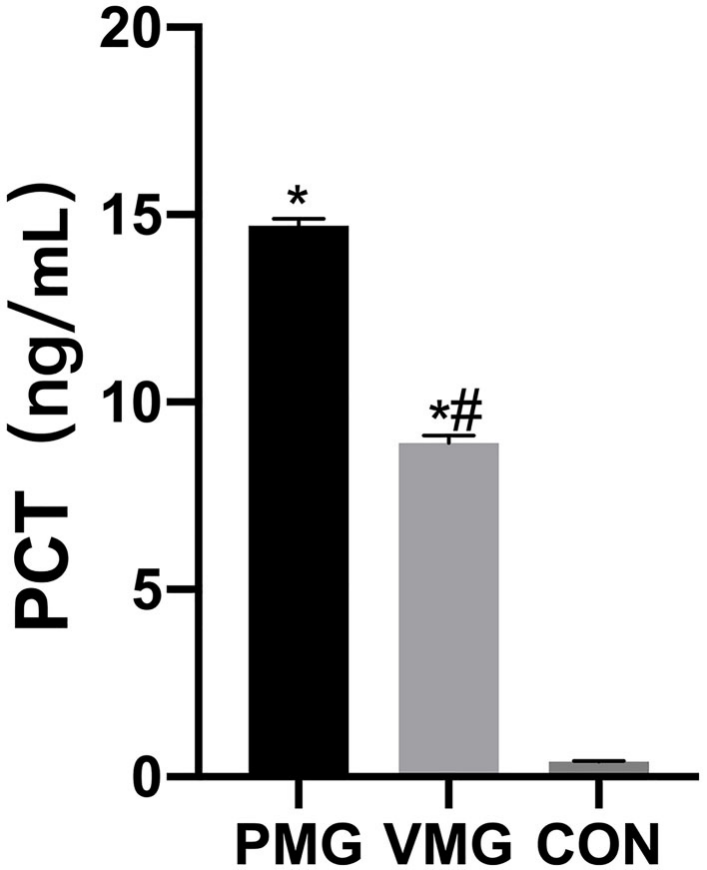
Comparison of serum procalcitonin (PCT) levels of children. VMG: viral meningitis group; PMG: purulent meningitis group; CON: control group. Data are reported as means±SD. *P<0.05, *vs* CON; ^#^P<0.05, *vs* PM (ANOVA).

**Figure 5:**
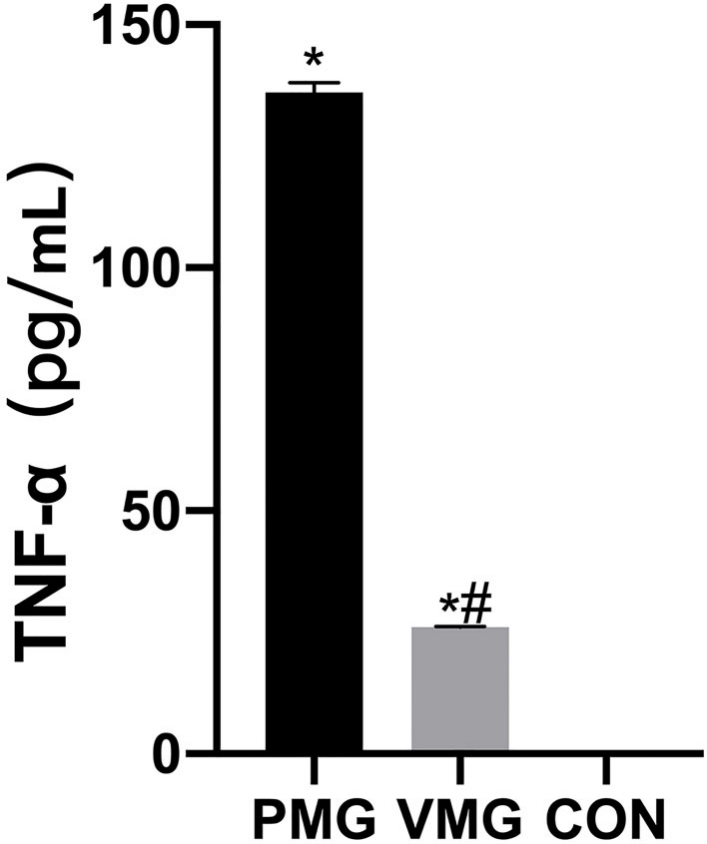
Comparison of serum tumor necrosis factor-α (TNF-α) levels of children. VMG: viral meningitis group; PMG: purulent meningitis group; CON: control group. Data are reported as means±SD. *P<0.05, *vs* CON group; ^#^P<0.05, *vs* PMG (ANOVA).

Serum CRP levels were significantly higher in the PM group than in the CON and VM groups (P<0.05, Figure 3). Serum PCT levels and TNF-α levels in the PM group were also higher than those in the CON and VM groups (P<0.05, Figures 4 and 5, respectively).

### Diagnostic value of HBP, CRP, PCT, and TNF-α for PM

**Figure 6:**
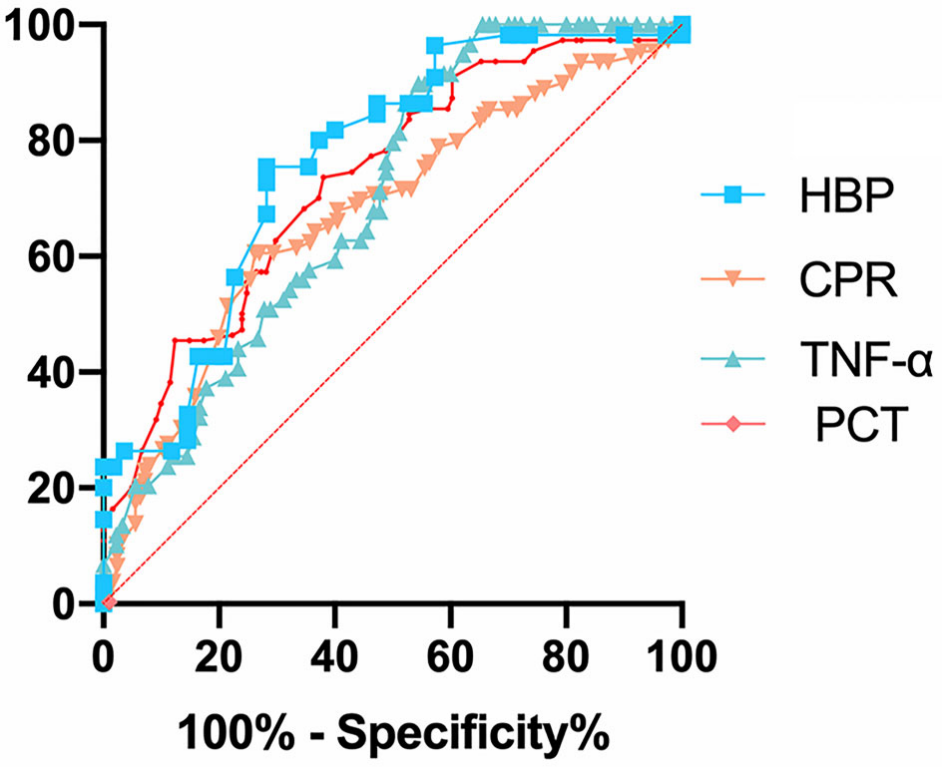
Receiver operating characteristic (ROC) curves for HBP, CRP, PCT, and TNF-α levels. HBP: heparin-binding protein; CRP: C-reactive protein; TNF-α: tumor necrosis factor-α; PCT: procalcitonin.

The diagnostic value of HBP, CRP, PCT, and TNF-α levels were assessed using ROC curves (Figure 6). The AUC of HBP was 0.8636, with an optimal cutoff value of ≥54.7 ng/mL, sensitivity of 98.3%, and specificity of 88.3%. The AUC of CRP was 0.6725, with an optimal cutoff value of ≥155.2 mg/mL, sensitivity of 55.6%, and specificity of 70.2%. The AUC of PCT was 0.7316, with an optimal cut-off value of ≥2.11 ng/mL, sensitivity of 68.2%, and specificity of 60.3%. The AUC of TNF-α was 0.6995, with an optimal cutoff value of ≥137.2 pg/mL, sensitivity of 58.7%, and specificity of 60.8%. HBP levels in the CSF had the highest diagnostic value among these four indices (Table 2).

**Table 2:** Levels of HBP, CRP, PCT, and TNF-α for the diagnosis of purulent meningitis.

Diagnostic markers	Critical value	Sensitivity (%)	Specificity (%)	AUC	95% AUC	P value
HBP (ng/mL)	54.7	98.3	88.3	0.8636	0.8007 to 0.9264	<0.0001
CRP (mg/mL)	155.2	55.6	70.2	0.6725	0.6030 to 0.7421	<0.0001
PCT (ng/mL)	2.11	68.2	60.3	0.7316	0.6677 to 0.7956	<0.0001
TNF-α (pg/mL)	137.2	58.7	60.8	0.6995	0.6174 to 0.7816	<0.0001

AUC: area under the receiver operating characteristic curve; HBP: heparin-binding protein; CRP: C-reactive protein; PCT: procalcitonin; TNF-α: tumor necrosis factor-α.

## Discussion

PM is one of the most common causes of neonatal mortality, with survivors often suffering from neurological impairment with severe sequelae due to delayed diagnosis, which impacts their learning abilities and has long-term consequences (1). The key to the treatment and prognosis of PM is early diagnosis. Our findings showed that HBP, CSF-WBC count, CRP, PCT, and TNF-α levels were all elevated in the children with PM. Moreover, HBP was more accurate for the diagnosis of PM than traditional diagnostic indicators and could be used as an early indicator of PM.

As an inflammatory disease, PM is mainly caused by a combination of inflammatory factors and pathogenic bacteria (12). Over the last decade, the rising misuse of antibiotics has resulted in higher incidences of antibiotic resistance in children (13), which has led to increased difficulty in the clinical diagnosis of PM, as well as VM.

As a serine protease, HBP acts as an important mediator of the inflammatory response (14). Evidence shows that HBP can be a good marker for the diagnosis of sepsis (15). Moreover, HBP tends to be elevated in patients with sepsis and accompanied by an exacerbation of the disease, compared to that in non-infected patients (16). It has been further shown (17) that elevated HBP levels (>30 ng/mL) are present in 80% of patients prior to the occurrence of severe sepsis. The results of a meta-analysis (18) demonstrated that HBP can be used as a serum biomarker to identify bacterial infections. Kandil et al. (19) concluded that HBP is involved in severe bacterial infections and, therefore, can be used as a potential diagnostic marker and therapeutic target for sepsis. They also reported that elevated HBP levels in the CSF can distinguish patients with acute bacterial meningitis from those with other central nervous system infections (19). However, this study only included a small number of cases. The present study showed a significant increase in HBP levels in the CSF of patients in the PM group compared to those in the VM and CON groups, which was consistent with the above results. In the strongest inflammatory response, leukocytes aggregated to the local site of infection, with enhanced monocyte activation and increased HBP secretion, resulting in elevated HBP levels in the CSF.

PCT, a newly discovered inflammatory factor, plays an important role in the diagnosis of infectious diseases (20). Serum PCT levels are relatively low under normal conditions. However, during bacterial infection, PCT expression increases (21). Davis et al. (22) showed that serum PCT levels in PM patients are higher than those in healthy controls (P<0.05). Our results showed that serum PCT levels were higher in the PM group than in the CON and VM groups (P<0.05). These findings suggested that inflammation can promote the secretion of PCT.

TNF-α is a common inflammatory factor, and under physiological conditions, its concentration is very low but increases dramatically during the onset of infection (23). TNF-α is a key player in the inflammatory response (24). In previous studies, TNF-α was shown to be significantly elevated in the serum of children with PM (25,26). We have shown that serum TNF-α levels in children with PM are elevated, which is highly consistent with published results. TNF-α is considered to induce the production of several cytokines.

There are some limitations to this study. This was a single-center clinical study that included a small number of cases. Therefore, further large-scale multicenter studies are needed for validation. In addition, serum HBP levels in children with PM were not included in the study, and the association between serum HBP levels and the severity of PM remains to be explored.

In conclusion, the results of the present study showed that children with PM had higher HBP levels in their CSF than patients with VM or without meningitis. Therefore, HBP levels in the CSF can be used as an index for the early diagnosis of PM.
